# European dimension of cannabinoid-like products use

**Published:** 2012-03-05

**Authors:** ME Surugiu, DG Mincă

**Affiliations:** “Carol Davila” University of Medicine and Pharmacy, Bucharest, 1-3 Dr. Leonte Street, District 5, Romania

**Keywords:** synthetic designer drugs, risk assessment, early warning system

## Abstract

The use of new psychoactive substances and the new patterns of drug use can have important public health and policy implications. Few countries have monitoring systems that are sensitive to this new phenomenon in the drug field, but methodological difficulties to detect them are considerable. Nonetheless, the importance of identifying potential new threats is widely recognized. The European Union’s Early-Warning System (EWS) provides a quick-response mechanism to the emergence of new psychoactive substances on the drug scene. The European Council’s decision on new psychoactive substances establishes a mechanism for the rapid exchange of information on new drugs. It also provides the possibility to trigger a formal risk assessment process, the findings of which may lead to a political decision to place new substances under control across the European Union.

**Abbreviations** EWS - Early-Warning System, THC – Tetrahydrocannabinol, EMCDDA - European Monitoring Center for Drugs and Drug Addiction

## Introduction

Since the establishment of the new early-warning system (EWS) in 1997 [**1**], more than 90 substances have been reported. Most of the new synthetic substances have stimulant properties, while only a few produce hallucinogenic effects. For the first time ever, in 2008, a synthetic cannabinoid (JWH-018, Naphthalen-1-yl-(1-pentylindol-3-yl) methadone) was reported through the early-warning system. The appearance of synthetic cannabinoids marks the latest stage in the development of designer drugs: from those based on fentanyl in the 1980s to ring-substituted phenethylamines in the late 1980s and tryptamines in 1990s, to piperazines and cathinone derivatives in the 2000s [**2**]. There are over a hundred compounds with cannabinoid receptor activity and it can be assumed that new substances from different chemical groups will continue to appear on the drug scene. All this presents a constant challenge to public health and law enforcement agencies.

### Synthetic Cannabinoids

Synthetic cannabis products are psychoactive herbal mixtures which have similar effects to those of cannabis, when inhaled. Based on patients’ clinical medical history, smoking these substances produces cannabinoid-like effects, although they do not contain THC (delta-9-tetrahydrocannabinol - the main psychoactive substance of cannabis plant) [**1**].

**Figure 1:**
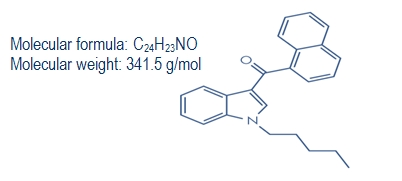
JWH-018 formula (1-pentyl-3-(1-naphthoyl) indole)

A large and complex variety of synthetic cannabinoids, most often cannabicyclohexanol, JWH-018, JWH-073 (naphthalen-1-yl-(1-butylindol-3-yl)methanone), CP 47 497 (2-[(1R,3S)-3-hydroxycyclohexyl]- 5-(2-methyloctan-2-yl)phenol), HU-210 (1,1-Dimethylheptyl-11-hydroxy-tetrahydrocannabinol) are used in an attempt to avoid the laws making cannabis illegal, thus conferring synthetic cannabis the status of a designer drug. Designer drugs are pharmaceuticals, created or reformulated to bypass current laws, by modifying the molecular structures of drugs (**Fig. 1**). The goal is to satisfy the users’ demand for drugs that can be obtained without prescriptions or other legal constraints [**2,4-5**].

Although synthetic cannabis does not produce positive results in drug tests for cannabis, it is possible to detect its metabolites in human urine. The synthetic cannabinoids contained in synthetic cannabis products have been declared illegal in many European countries. 

### Availability of Spice products

A European survey that took place in 2008, conducted by the European Monitoring Center for Drugs and Drug Addiction (EMCDDA), identified Spice products in 21 out of the 30 participating countries. For the purpose of the survey, “identification” means that these types of products were available in some form in the country. JWH or CP compounds were identified by forensic/toxicological analysis in 8 out of 21 countries where Spice products were available: Austria, Germany, Finland, France, Hungary, Poland, Slovenia and the United Kingdom. The substances identified were: JWH-018; CP 47 497 - C8 homologue; CP 47 497; JWH-073 (naphthalen-1-yl-(1-butylindol-3-yl) methanone) [**2**].

### The composition of Spice products

The products marketed on the internet and in specialized shops under the names “Spice”, "Smoke", "Scence", "Yucatan Fire", or "Skunk" and advertised as an exotic incense blend which releases a rich aroma, along with the disclaimer not for human consumption, have been widely reported as producing many of the physiological effects of marijuana. 

The labels on the packages indicate that the products are composed of as many as 14 ingredients of plant origin, with every package weighting between 0.4–3.0 g. Synthetic ingredients are not mentioned in the product information. 

While at least two of the ingredients — Pedicularis densiflora and Leonotis leonurus — may have some psychoactive effect, little is known about the pharmacology and toxicology of the plant materials purportedly contained in spice products (**Table 1**). All of these products contain differing varieties of herbs and other botanicals [2-3].

**Table 1:** Herbal components of Spice products

Common name	Species	Family
Beach bean	Canavalia maritima, syn. C rosea	Fabaceae
White and blue water lily	Nzmphaea alba and N. caerulea	Nymphaeaceae
Dwarf skullcap	Scutellaria nana	Lamiacae
Indian warrior	Pedicularis densiflora	Orobanchaceae
Lion’s ear/tail, Wild dagga	Leonotis leonuru	Lamiacae
Maconha brava’	Zornia latifolia or Z. diphylla	Fabaceae
Blue/Sacred lotus	Nelumbo nucifera	Nelumbonaceae
Honeyweed/Siberian motherwort	Leonurus sibiricus	Lamiaceae
Marshmallow	Althaea officinalis	Malvaceae
Dog rose/Rosehip	Rosa canina	Roseceae

In 2008, spice products, as well as various other spice-like herbal mixes, could be purchased from online shops, and were available in specialized shops selling “legal highs” in at least nine EU Member States (Czech Republic, Germany, Latvia, Lithuania, Luxembourg, Austria, Poland, Portugal, Romania, United Kingdom). Without a legislative framework to control its distribution and insufficient detection methods, the law enforcement agencies faced a significant challenge in addressing the problem of synthetic cannabinoids. 

Extensive forensic investigations have been undertaken by EU Member States in order to identify the psychoactive ingredients of spice products. In December 2008, Germany and Austria detected the synthetic cannabinoid JWH-018. The chemical structure of JWH-018 differs substantially from that of tetrahydrocannabinol (THC), the main active principle in all cannabis products. In experimental animals, JWH-018 produces the same effects as THC and has been reported to be more potent. Earlier, in 2009, a second synthetic cannabinoid named CP 47 497, and three of its homologues were detected in spice samples in Europe. Some reports indicate that JWH-018 binds to the CB1 receptor (cannabinoid receptor) with even greater affinity than marijuana [**3**]. 

### Effects of synthetic cannabinoids

The reported pharmacological effects of synthetic cannabinoids smoked are very similar to that of marijuana. This comes as no surprise, given that Spice and K2 are THC agonists - meaning they chemically bind to the same brain receptor (CB1) and trigger many similar responses as marijuana. The physiological effects of synthetic cannabinoids include: increased heart rate and blood pressure, altered state of consciousness, perceptual alterations (time distortion), intensification of sensory experiences, impaired short-term memory [**7**].

### Laws regarding synthetic cannabinoids

Responding to potential health concerns, the Member States have taken diverse legal actions to ban or otherwise control spice products and related compounds (**Table 2**). Germany used its emergency legislation for narcotics to control for one year five synthetic cannabinoids found in spice products. France classified as narcotics six synthetic cannabinoids found in spice products. Austria used its medicines act to prohibit smoking mixes containing six synthetic cannabinoids from being imported or marketed in the country. Luxembourg decided to control various synthetic cannabinoids as psychotropic substances. Poland amended the narcotic law, placing under control JWH-018 and two of the claimed herbal ingredients of spice [**2**].

**Table 2:** Legislative measures for incriminating synthetic cannabinoid at European level

Country	Legislative measures
Austria	On the 18th of December 2008, the Austrian Ministry of Health announced that Spice would be controlled under Paragraph no. 78 of Austrian drug law, on the grounds that it contains an active substance which affects the functions of the body, and the legality of JWH-018 is under review
Germany	JWH-018, CP 47,497 and the C6, C8 and C9 homologues of CP 47,497 are illegal in Germany since the 22nd of January 2009
Finland	Spice blends are classified as a medicine in Finland and, therefore, it is illegal to order them without a prescription
France	JWH-018, CP 47,497 (and its homologues) and HU-210 were all made illegal in France on the 24th of February 2009
Ireland	From June 2010, JWH-018, along with a variety of other designer drugs have been declared illegal
Latvia	JWH-018, JWH-073, CP 47,497 (and its homologues) and HU-210 are all banned in Latvia as well as leonotis leonurus
Poland	JWH-018 and many of the herbs mentioned on the ingredient lists of Spice and similar preparations were made illegal in May 2009
Romania	Spice was made illegal in Romania on the 15th of February 2010, Governmental Ordinance no. 6/2010
Sweden	CP 47,497-C6, CP 47,497-C7, CP 47,497-C8, CP 47,497-C9, JWH-018, JWH-073 and HU-210 were all made illegal in Sweden on the 15th of September 2009
United Kingdom	Spice was legal in the United Kingdom until December 2009, when it was classified as a Class B drug

## Conclusions

Spice and other herbal products are often referred to as legal highs. It appears that most of the ingredients listed on the packaging are actually not present in the Spice products and it is seems likely that the psychoactive effects reported are most probably due to added synthetic cannabinoids, which are not shown on the label. Different amounts or combinations of these substances seem to have been used in different Spice products to produce cannabis-like effects. It is possible that the substances from these or other chemical groups, with a cannabinoid agonist or other pharmacological activity could be added to any herbal mixture. So far, the Spice and Spice-like preparations in Europe have been found to contain at least nine new substances from three chemically distinct groups of synthetic cannabinoids (JWH, CP and HU). This presents an ongoing challenge, not only for their forensic and toxicological identification, but also for risk assessments and the development of possible control strategies.
